# Human ossicular-joint flexibility transforms the peak amplitude and width of impulsive acoustic stimulia)

**DOI:** 10.1121/1.5039845

**Published:** 2018-06-07

**Authors:** Peter K. Gottlieb, Yona Vaisbuch, Sunil Puria

**Affiliations:** Department of Mechanical Engineering, Stanford University, Stanford, California 94305, USA; Department of Otolaryngology–Head and Neck Surgery, Stanford University, Stanford, California 94305, USA; Department of Otolaryngology, Eaton-Peabody Laboratories, Massachusetts Eye and Ear Infirmary, Harvard Medical School, 243 Charles Street, Boston, Massachusetts 02114, USA

## Abstract

The role of the ossicular joints in the mammalian middle ear is still debated. This work tests the hypothesis that the two synovial joints filter potentially damaging impulsive stimuli by transforming both the peak amplitude and width of these impulses before they reach the cochlea. The three-dimensional (3D) velocity along the ossicular chain in unaltered cadaveric human temporal bones (*N* = 9), stimulated with acoustic impulses, is measured in the time domain using a Polytec (Waldbronn, Germany) CLV-3D laser Doppler vibrometer. The measurements are repeated after fusing one or both of the ossicular joints with dental cement. Sound transmission is characterized by measuring the amplitude, width, and delay of the impulsive velocity profile as it travels from the eardrum to the cochlea. On average, fusing both ossicular joints causes the stapes velocity amplitude and width to change by a factor of 1.77 (*p* = 0.0057) and 0.78 (*p* = 0.011), respectively. Fusing just the incudomalleolar joint has a larger effect on amplitude (a factor of 2.37), while fusing just the incudostapedial joint decreases the stapes velocity on average. The 3D motion of the ossicles is altered by fusing the joints. Finally, the ability of current computational models to predict this behavior is also evaluated.

## I. INTRODUCTION

Historically the middle ear of terrestrial vertebrates has been understood to serve a simple purpose: to act as an impedance transformer to maximize the transfer of acoustic energy from the environment into the specialized mechanotransduction organs in the fluid-filled cochlea (e.g., Helmholtz, 1912; Wever *et al.*, 1948; Wever and Lawrence, 1950). This impedance transformation is largely accomplished by the area ratio between a large tympanic membrane (TM) and a smaller cochlear opening. In the cases of most terrestrial vertebrates, the TM is connected to the cochlea via a simple piston-like structure known as the columella. In mammals, however, a more complicated chain of three distinct ossicles—the malleus, incus, and stapes—connected by flexible joints, replaces the columella (e.g., Mason and Farr, 2013). Since the simple columella system seems to satisfy the main goal of the middle ear—impedance matching—the functional implications of the mammalian three-ossicle system have been long debated. Of particular interest is the flexibility imparted to the mammalian middle ear by the ossicular joints.

The two ossicular joints, known as the incudomalleolar joint (IMJ) and incudostapedial joint (ISJ) have very different structures. In humans, the IMJ has been described as a diarthrodial joint with a saddle-shaped articulation surface. The joint capsule is filled with synovial fluid and surrounded by a layer of fibrous tissue (Marquet, 1981; Sim and Puria, 2008). The ISJ is also a synovial joint, though the geometry differs from the IMJ. The long process of the incus terminates in the lenticular process, which consists of a thin, relatively flexible stem connected to a flat, plate-like cap. The cap of the lenticular process and the head of the stapes form the articular surfaces of the ISJ, which are again surrounded by a synovial fluid-filled joint capsule (Karmody *et al.*, 2009). The relative flexibility and function of these two joints has been a controversial topic in the literature.

Early studies of the IMJ have concluded that it is functionally immobile at normal sound-pressure levels (Kirikae, 1980), and Elpern *et al.* (1965) find that immobilizing the IMJ has a negligible effect on sound transmission, though they only measure up to 4 kHz. As research has progressed, others have found that the IMJ displays a frequency-dependent mobility, becoming more mobile at higher frequencies (Guinan and Peake, 1967; Willi *et al.*, 2002). Contemporary work has supported the idea that the IMJ is mobile at frequencies above 2 kHz (Gerig *et al.*, 2015), as well as under quasi-static loading conditions (Ihrle *et al.*, 2016). The ISJ has received relatively less scrutiny than the IMJ, though a number of studies have demonstrated that it is functionally mobile at hearing frequencies (Nakajima *et al.*, 2005; Zhang and Gan, 2011) and that fixing it causes changes in sound transmission (Hato *et al.*, 2003; Alian *et al.*, 2013).

While the general understanding of ossicular-joint mechanics is still evolving, a number of hypotheses concerning possible evolutionary advantages conferred by their potential flexibility, such as the ability to hear high frequencies (Hemilä *et al.*, 1995; Puria and Steele, 2010) or protect the cochlea from damage due to high static-pressure buildup in the ear canal by decoupling the stapes from the TM (Hüttenbrink, 1988), have previously been presented in the literature. Perhaps most interesting is the proposition that the complicated mammalian middle ear provides mechanisms to protect sensitive cochlear structures from damage. This idea is particularly attractive because, in contrast to mammals, other terrestrial vertebrates with columella-type middle ears have the ability to repair and regenerate cochlear hair cells. Protecting the mammalian cochlea from damaging input may therefore be a critically important evolutionary pressure. This present work aims to extend the idea of the protective middle ear beyond static and into transient pressures by proposing a new hypothesis that the ossicular joints filter potentially damaging impulsive stimuli by transforming both the peak amplitude and width of these impulses before they reach the cochlea.

The adverse effects of impulsive acoustic stimuli on the hearing system have been under active investigation since at least the 1940s, when researchers began to examine the relationship between various types of gunfire and temporary deafness (Davis *et al.*, 1946; Reid, 1946). More recently, a significant effort has been expended in attempts to develop an accurate algorithm for assessment of the relative hazard of various impulses (e.g., Price, 1983, 1986; Patterson, 1991; Price, 2007). Even further work has examined the mechanisms of hearing loss due to blast trauma from the outer and middle ear (e.g., Gan *et al.*, 2016; Walilko *et al.*, 2017) to the cochlea (Cho *et al.*, 2013; Greene *et al.*, 2016, 2017). Clearly, understanding the effects of these impulsive stimuli is an important goal with straightforward relevance to public health. Despite this, little work has been done to quantify the way in which these stimuli are transformed by the middle ear as they propagate from the environment into the cochlea. The passive effect of the middle ear on impulsive stimuli is also of particular interest, as the middle-ear acoustic reflex, which triggers the potentially protective contraction of the middle-ear muscles, operates too slowly to have an effect in these cases.

While the previously discussed studies have characterized the behavior of the ossicular joints under certain conditions, none to our knowledge have yet directly examined the transmission of transient signals through the ossicular chain. In this study, laser-Doppler vibrometry is used to measure the vibratory response of the ossicles in cadaveric human temporal bones stimulated with realistic acoustic impulses directly in the time domain. The impact of the IMJ and ISJ on sound transmission is individually characterized through selective fusion of the joints, and these effects are quantified by calculating the relative motion of selected points on the ossicles from the umbo to the stapes. Further, by using a three-dimensional (3D) laser-Doppler vibrometer (LDV), no *a priori* assumptions about the ossicular vibration patterns are necessary.

Computational models play an important role in middle-ear research. Experiments that would be impossible in the lab can be done easily “in silico,” and future devices in the design stage can be tested quickly without the need for lengthy and expensive temporal-bone studies. In order to be useful as tools for studying middle-ear behavior, though, they must be able to predict behaviors beyond those with which they have been tested. Therefore, after completing the experimental measurements of impulse transmission through the middle ear, two previously published computational models are evaluated for consistency with these new results. Both an abstract circuit model and a detailed finite element (FE) model are chosen. Both models have originally been developed by validating their responses against measurements of steady-state sound transmission in healthy ears over the frequency range of human hearing. The results of this comparison suggest that while these models accurately represent some aspects of the transient middle-ear behavior, additional work is required to improve both types of models.

## II. METHODS

### A. Materials

Thirteen fresh adult temporal bones are used in this study, obtained from Anatomy Gifts Registry (Hanover, MD). The bones are cut to about 3 in. on a side and stripped of the skin and pinna at the time of harvest, preserving the external ear canal and all middle- and inner-ear structures. All are from donors with no history of otologic disease, and the specimens are visually screened for middle-ear pathologies upon arrival. Measurements have been successfully completed in nine bones from seven female and two male donors. The average age at death is 58.3 yrs (range 27–76). The temporal bones are kept refrigerated and wrapped in saline-soaked gauze until the time of use.

### B. Sample preparation

After inspecting the bones for visible middle-ear defects, such as a perforated TM, any remaining soft tissues are removed. If at any point the TM, ossicles, or supporting structures are damaged during these procedures, the experiment is halted and the temporal bone is discarded. A Medtronic Visao high-speed otologic drill is used to expose the facial recess through the mastoid. In order to provide the large field of view necessary for this experiment, the facial recess is extended as much as possible without drilling into the lateral semicircular canal or damaging the tympanic annulus. The horizontal and vertical segments of the facial nerve are also removed, and the air cells from the tegmen mastoideum are drilled away to allow access to the IMJ. Following this, the anterior wall of the external ear canal is drilled down to a small bony rim surrounding the tympanic annulus in order to expose the TM to free-field acoustic stimulation. The posterior wall of the ear canal is left intact to provide an acoustic barrier between the exposed middle-ear cavity and the TM.

**FIG. 1.**
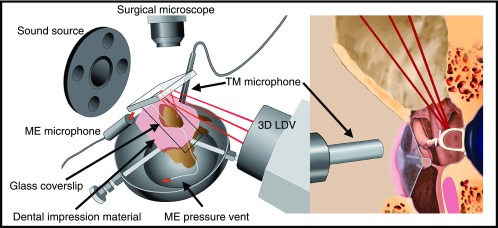
Experimental setup. Left: Overview of the experimental setup. The temporal bone is held in a standard temporal-bone bowl. The mastoid opening is covered with dental-impression material (pink) and acoustically sealed with a glass coverslip. Two Etymotic Research ER-7C probe tubes are inserted into the cavity through the dental impression material. One is connected to the ME microphone, and the other is left open as a pressure vent. The lasers from the 3D LDV are reflected into the mastoid opening by a dichroic mirror, and the opening is visualized through the mirror by a surgical microscope. The sound source is used to play acoustic impulses, and the pressure at the TM is measured using the TM microphone. Right: Close-up of the ear-canal and middle-ear-cavity openings. The three laser beams from the LDV are focused on the ossicles through the mastoid opening. The tip of the TM microphone is placed 1–2 mm from the lateral surface of the TM.

After completing the dissection, the middle-ear cavity is gently stuffed with damp cotton and the entire mastoid opening is covered with a dental alginate impression material (Jeltrate). The Jeltrate mixture is made with a slightly higher ratio of water to powder than suggested by the instructions in order to make it easier to mold around the specimen and seal the mastoid opening. In order to create a flat surface that would be easy to seal with a glass coverslip, the Jeltrate is molded around the temporal bone by first mounding the wet mixture on the lab bench and then inserting the temporal bone into the material with the mastoid opening facing down. A spatula is then used to mold the Jeltrate around the rest of the specimen. Special care is taken to keep the Jeltrate away from the TM. After the Jeltrate is allowed to set, a scalpel is used to carefully cut a hole in the vicinity of the mastoid opening, and the cotton is removed. Small grooves are cut into the surface of the Jeltrate adjacent to the opening in order to accommodate two Etymotic Research ER7-14C probe tubes. One tube is connected to the ER-7C microphone to monitor the acoustic leakage into the middle-ear cavity, while the other functions as a high-impedance pressure outlet to prevent static-pressure buildup. Retroreflective glass beads (*D* = 30–53 μm) are placed on the ossicles using a fine paintbrush (Artetje 630 Camlon Pro Plata 100/0). A small piece of dry cotton is placed in a corner of the cavity to regulate humidity and prevent fogging. Finally, a glass slide is placed over the opening and affixed with Vaseline to create an acoustically sealed window into the middle-ear cavity (see Fig. 1). Any remaining parts of the temporal bone still exposed to air are wrapped in plastic film to prevent drying, and the bone is placed in a standard temporal-bone holder.

### C. Measurement system

SyncAv (v0.34) is a custom LabVIEW (National Instruments, Austin, TX) based data-acquisition program that performs synchronous averaging between up to two output and six input channels. This software runs on a National Instruments PXI data acquisition computer with a maximum sampling rate of 204.8 kHz. For this experiment, a sampling rate of 120 kHz, 8192-sample buffer length, and 10 averages are used in order to provide a temporal resolution high enough and noise floor low enough to capture the relevant transient vibrations. A Polytec (Waldbronn, Germany) CLV-3D LDV system, mounted on three motorized and computer-controlled linear translation stages, allows the specimen to stay fixed while only the laser is moved to focus on different locations. SyncAv is used to control the motion of these stages and record their positions for each measurement location with 6.25-*μ*m resolution. The three LDV lasers are reflected into the specimen by a dichroic mirror in order to allow visualization of the experimental field by a Zeiss OPMI-1 operating microscope (Fig. 1). A ThorLabs (Newton, NJ) CMOS USB camera (part no. DCC3240C) connected to the microscope is used to monitor and capture images of the specimens during measurements.

Sound pressure at the TM is measured using a Sokolich G-II ultrasonic probe microphone system, which has a flat frequency response up to 65 kHz. The tip of the rigid probe tube is placed within 2 mm of the TM. Pressure inside the middle-ear cavity is simultaneously measured using an Etymotic ER-7C microphone connected to the soft probe tube inserted through the Jeltrate material (Fig. 1).

**FIG. 2.**
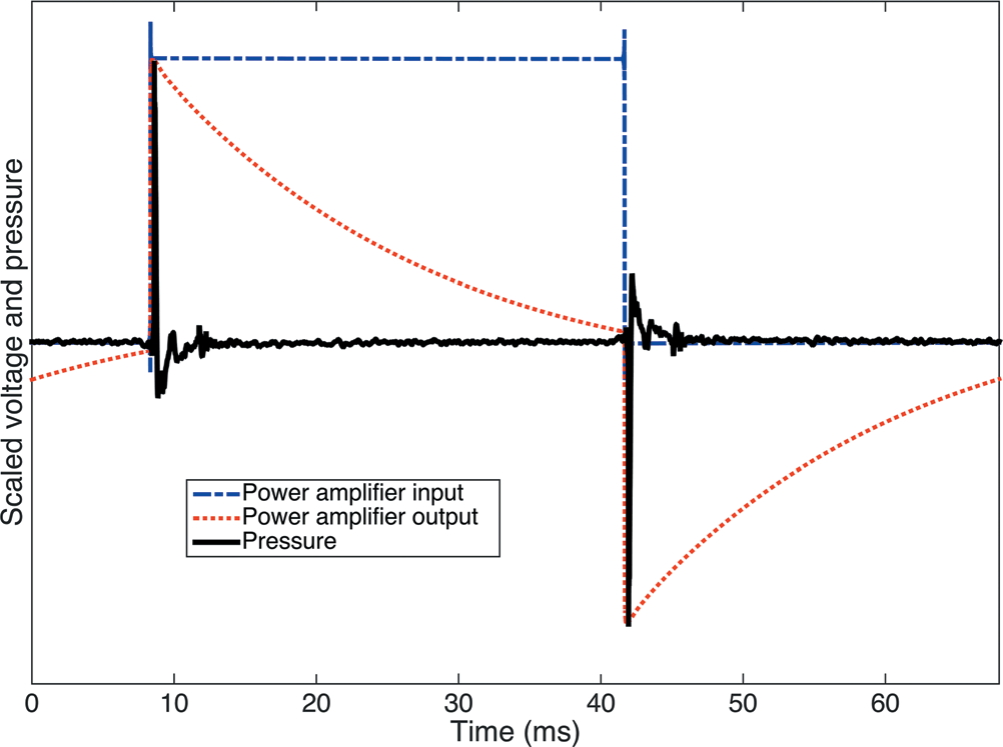
Acoustic impulse generation. The SyncAv software is used to generate a square pulse (blue, dashed line), which is fed into a power amplifier. The output of the power amplifier (red, dotted line) is then sent to an ultrasonic tweeter, which generates the acoustic impulses (black, solid line). The entire stimulus is offset in time to avoid artifacts from signal aliasing.

An ultrasonic tweeter (Tymphany, Sausalito, CA) presents the acoustic impulses. SyncAv is used to generate a square pulse with a duration of 4000 samples (33.3 ms), which is passed through a GX3 power amplifier (QSC, Costa Mesa, CA) before reaching the tweeter (see Fig. 2). The square pulse is offset by 1000 samples (8.3 ms) to minimize time-aliasing effects. The near-instantaneous change in voltage at the leading and tailing ends of the square pulse cause impulsive displacements of the tweeter diaphragm, resulting in paired positive and negative acoustic impulses. The impulses have a peak pressure of 20 Pa (equivalent to roughly 120 dB sound pressure level) and a full width at half maximum (FWHM) of about 0.1 ms.

### D. Measurement protocol

**FIG. 3.**
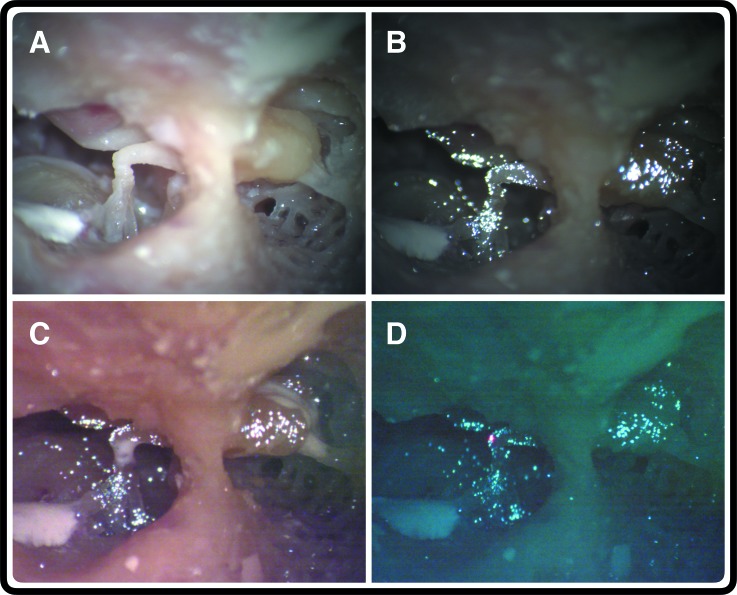
Images of specimen preparation. (A) The view of the fully dissected middle-ear cavity through the surgical microscope in the orientation used during measurements. Note that the umbo, malleus head, IMJ, incus body and long process, ISJ, and stapes are all visible. The intact incus buttress is seen in the foreground. The white patch on the bottom left is a drop of dental cement that has been used to reinforce the pyramidal process, which has been drilled very thin during this dissection. (B) The same specimen with retroreflective glass beads applied. (C) The specimen after both joints have been fused with dental cement. (D) The three laser beams from the 3D LDV (red dot) are focused on the tip of the incus long process. The image has a green cast since it is taken through the dichroic mirror.

For each specimen, at least 10 points on the ossicles are identified as desired measurement locations. Typically, this includes at least two near the umbo and along the manubrium, two on the malleus head, three to four on the incus body, two on the incus long process and near the lenticular process, and one to three on the stapes posterior crus. The orientation of the specimen is adjusted to allow access by the 3D LDV to all of the points with a single frame of reference, and the temporal-bone holder is fixed in place using modeling clay. The coordinates of the measurement points are obtained and saved by SyncAv with a resolution of 6.25 *μ*m. This allows the software to automatically and immediately return and refocus the 3D LDV on any of the previously measured points (see Fig. 3).

Before proceeding with the impulse measurements, sound stimuli consisting of 80 pure tones logarithmically spaced from 100 Hz to 20 kHz are presented using the ultrasonic tweeter while the 3D LDV records the vibration velocity at the stapes. Stapes velocity is compared to an ASTM standard for temporal-bone models (Rosowski *et al.*, 2007), and temporal bones with responses that deviate significantly from this standard are discarded. This was the case for two bones. Following this verification, the 3D LDV is focused on each of the previously identified measurement locations in turn while impulsive acoustic stimuli are presented.

Following the baseline measurements, the glass coverslip is removed and a small drop of Durelon dental cement (3M, Maplewood, MN) is placed on either the IMJ or the ISJ using a Rosen needle pick [see Fig. 3(C)]. The IMJ is fused first in five temporal bones, while the ISJ is fused first in the remaining four. Care is taken not to move the sample or displace any of the retroreflective beads during this process. The coverslip is then replaced and the cement is allowed to set for roughly 15 min. Joint fusion is visually confirmed by removing the coverslip and gently palpating the ossicles with a Rosen needle. The lateral surface of the TM is briefly moistened with normal saline, and measurements are then repeated on the fused specimen. Finally, the remaining joint is fused and measurements are repeated again. The vibrational velocity of the cochlear promontory is also collected as a measure of experimental artifact. If the middle-ear structures or TM are damaged during this process, the experiment is halted and the specimen is discarded. This was the case for one specimen, for which the experiment had to be stopped after measuring the fused-ISJ case.

### E. Data analysis

**FIG. 4.**
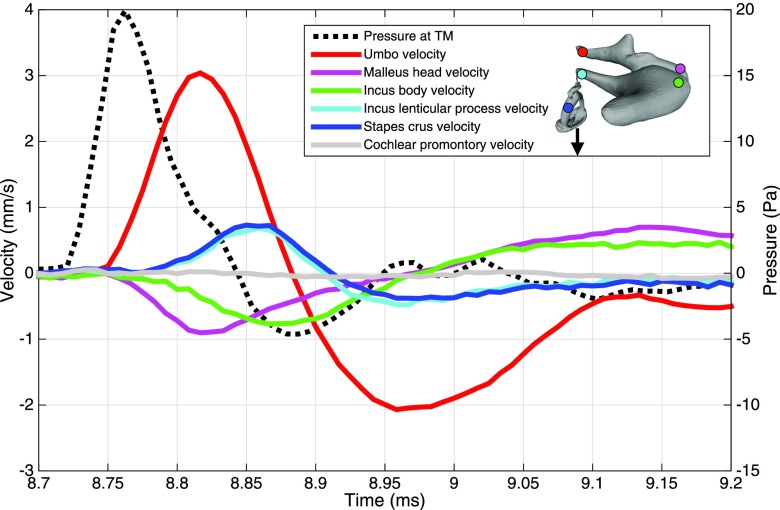
Summary of impulse transmission in a representative specimen. The velocities in the footplate-normal direction (shown by the arrow), measured at five points on the ossicles as well as on the cochlear promontory, are shown (solid lines). The colored dots on the ossicle model correspond to the approximate measurement locations. Note how the velocities at the malleus head (magenta) and incus body (green) are inverted relative to the other curves due to the rocking motion of the ossicles. The delay due to the IMJ is clearly visible between these two curves. The pressure at the TM is also shown (dotted line), with units given by the *y*-axis on the right. The time on the *x* axis is given referenced to the beginning of the measurement window, and the stimulus is offset as seen in Fig. 2.

Data analysis is performed in Matlab (Mathworks, Natick, MA). The SyncAv Toolbox, a custom set of scripts designed to interface with SyncAv-generated files, is used to organize the data and for visualization of results. The three Cartesian components of the time-domain velocity profile at five representative points across the ossicles are plotted first. These points include the umbo, malleus head, incus body, incus lenticular process, and stapes posterior crus, and analogous points are chosen for each specimen (see Fig. 4, inset). After noting that the velocity in the “piston,” or stapes-normal, direction is the dominant motion in all cases, subsequent analysis is restricted to this component of velocity.

**FIG. 5.**
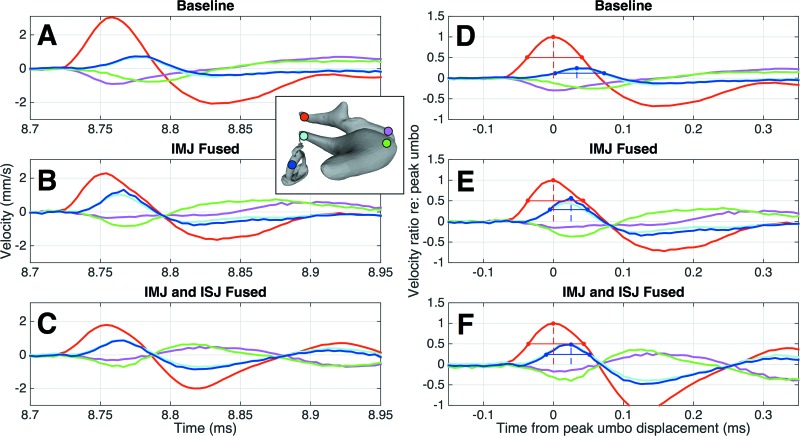
Effects of joint fusion. (A)–(C): The velocities at five points on the ossicles in a representative specimen are shown in the baseline (A), IMJ fused (B), and IMJ and ISJ fused (C) cases. The line colors correspond to the colors of the dots on the inset image of the ossicles. (D)–(F): The same velocities are shown after normalizing to the umbo curve. All velocities are scaled by the peak umbo velocity, and shifted so that the peak umbo velocity occurs at time *t* = 0. The lines on the umbo and stapes curves show the calculation of the amplitude (dashed line) and the FWHM (solid line) of each curve. These metrics are later used to quantify the effects of joint fusion and compare between different specimens.

**FIG. 6.**
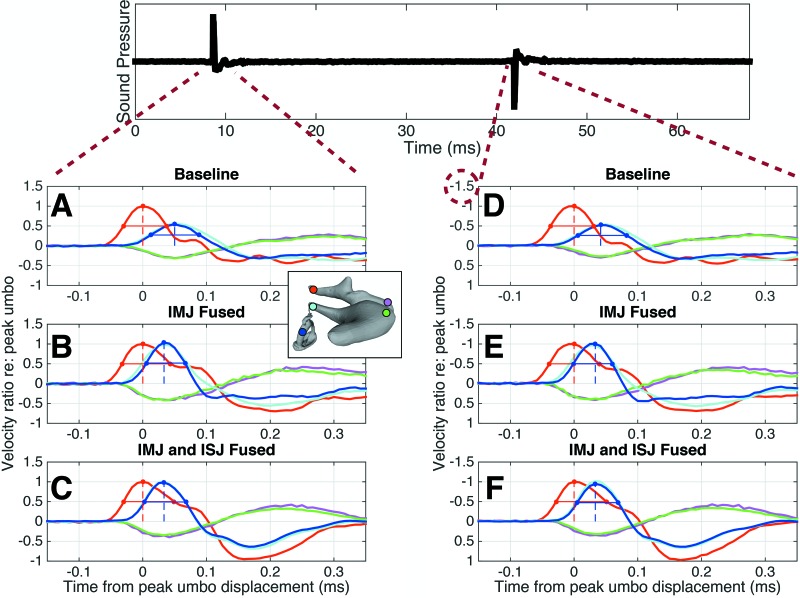
Ossicular response to positive and negative pressure impulses. Top: the full pressure stimulus used during the experiment is shown. The peak pressures are about 20 Pa. (A)–(C): the ossicular response to the positive pressure impulse is shown in a representative specimen, for the baseline (A), IMJ fused (B), and both joints fused (C) cases. (D)–(F): the ossicular responses to the negative pressure impulse are shown in the same specimen. Note that the *y*-axis has been inverted to facilitate comparisons between the responses to positive and negative pressures. The line colors correspond to the colors of the dots on the inset image of the ossicles.

In order to compare the impulse transmission through the ossicles across different specimens quantitatively, the time-domain velocities are normalized by the umbo-velocity profile. First, the peak umbo-velocity amplitude and location in time is calculated. Then, all velocities are divided by this peak velocity, and shifted such that the peak umbo velocity occurs at time *t* = 0. Next, the FWHMs of the scaled umbo- and stapes-velocity profiles are calculated, along with the amplitude and location of the peak scaled stapes-crus velocity. This process is repeated for each fusion case (see Fig. 5). Since each measurement includes both positive and negative pressure impulses, the velocity response is checked for symmetry with respect to stimulus polarity (see Fig. 6). Following this, all subsequent analyses are reported only for the response to the first, positive, impulse since the negative impulse provides no additional information due to the response symmetry.

**FIG. 7.**
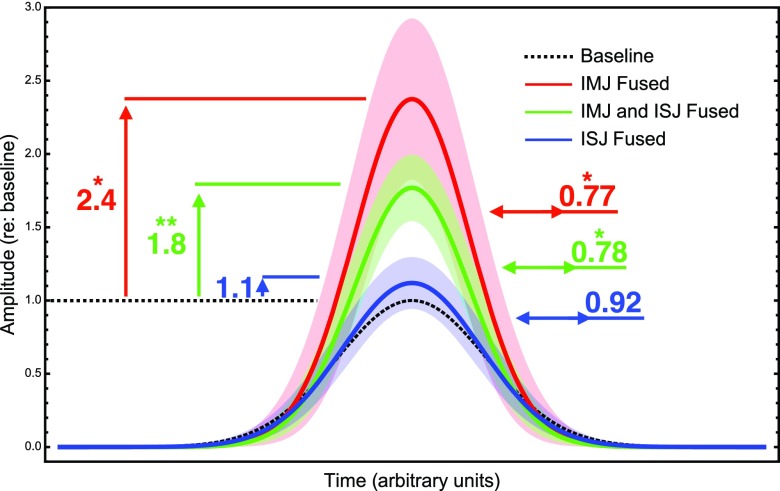
Average effects of joint fusion on impulse transmission. The average transformation in pulse amplitude and width resulting from fusing the IMJ only (red), IMJ and ISJ (green), and ISJ only (blue) is applied to a generic impulse with amplitude 1 and arbitrary width (dashed line, black). The numbers with the vertical and horizontal arrows show the average values of the amplitude and width changes, respectively. Significance at the *p* < 0.05 and *p* < 0.01 levels is shown with one and two stars, respectively. The shaded areas show the standard error in the measurements.

Finally, the change in scaled stapes peak amplitude and width resulting from joint fusion is calculated for each specimen, and the mean and standard deviation are found across all specimens (see Table I). A single-sided Student's *t*-test is performed on the mean amplitude and width changes to check for statistical significance. The average ossicular delay is also calculated. For visualization purposes, the mean change in stapes-velocity profile (amplitude and width) resulting from joint fusion is also applied to a generic “prototype impulse.” This prototype impulse has been chosen to be a simple Gaussian with an amplitude of one and arbitrary width (see Fig. 7).

**TABLE I.** Summary of measured changes in stapes velocity after joint fusion. The average change in the amplitude and FWHM relative to the baseline case is shown (dimensionless) for all of the experimental fixations, along with the standard error. The *p*-values have been calculated using a one-sided Student's *t*-test.

Joint(s) fused	Stapes velocity **amplitude** ratio	Stapes velocity **width** ratio
Baseline → IMJ (*n* = 5)	2.374 ± 0.550 (***p* = 0.0335**)	0.7707 ± 0.0775 (***p* = 0.0208**)
Baseline → ISJ (*n* = 4)	1.119 ± 0.177 (*p* = 0.3)	0.9211 ± 0.0765 (*p* = 0.1891)
Baseline → IMJ + ISJ (*n* = 8)	1.769 ± 0.226 (***p* = 0.0057**)	0.7821 ± 0.0745 (***p* = 0.0111**)
IMJ → IMJ + ISJ (*n* = 5)	0.7334 ± 0.0968 (***p* = 0.0256**)	1.1474 ± 0.1089 (*p* = 0.1238)
ISJ → IMJ + ISJ (*n* = 3)	1.780 ± 0.181 (***p* = 0.0249**)	0.6924 ± 0.1417 (*p* = 0.0810)

### F. 3D motion reconstruction

For visualization purposes, the 3D motion of the ossicles is reconstructed from the pointwise 3D-velocity measurements using a least-squares fit to calculate the rigid-body motion of each ossicle. Since this technique requires at least three distinct measurement locations on each ossicle, with reasonable spacing between them, it is only possible to use it on a single specimen (TB13), for which enough measurements have been obtained on the stapes.

The 3D-velocity $\vec{v}$ of a point p on a rigid body can be described by

$${\vec{v}}_{p}={\vec{v}}_{O}+\vec{\omega }\times {\vec{r}}_{p},$$ (1)

where ${\vec{v}}_{O}$ is the linear velocity of a reference point O (in this case chosen to be the center of mass of the ossicle), $\vec{\omega }$ is the angular velocity of the rigid body, and $\;{\vec{r}}_{p}$ is the vector (x,y,z) from O to p. In matrix form, this reduces to

$${\vec{v}}_{p}=A{\vec{v}}_{r},$$ (2)

where

$$\underline{\underline{A}}=\left[\begin{array}{cccccc} 1 & 0 & 0 & 0 & z & -y\\ 0 & 1 & 0 & -z & 0 & x\\ 0 & 0 & 1 & y & -x & 0 \end{array}\right],\;\text{and}\;{\vec{v}}_{r}=\left\{\;\begin{array}{c} v_{Ox}\\ v_{Oy}\\ v_{Oz}\\ \omega_{x}\\ \omega_{y}\\ \omega_{z} \end{array}\right\}.$$ (3)

Now, for each n measured points on an ossicle, there is a known velocity ${\vec{v}}_{mn}$. If the ossicle is considered to be a rigid body, the following relationship must therefore hold:

$$\left\{\begin{array}{c} {\vec{v}}_{m1}\\ {\vec{v}}_{m2}\\ \vdots \\ {\vec{v}}_{mn} \end{array}\right\}=\left[\begin{array}{c} A_{\;1}\\ A_{\;2}\\ \vdots \\ A_{\;n} \end{array}\right]{\vec{v}}_{r}.$$ (4)

If Eq. (4) is solved for the rigid-body velocity components vector ${\vec{v}}_{r}$, the linear velocity of any point on the body can be calculated using Eq. (2). Since the ossicles are not truly rigid and the measurement system is noisy, however, this relationship does not strictly hold and an approximation must be used. This has been done using the standard least-squares solution,

$${\vec{v}}_{r}={\left(\;A^{T}A\;\right)}^{-1}\left(\;A^{T}{\vec{v}}_{m}\right).$$ (5)

**FIG. 8.**
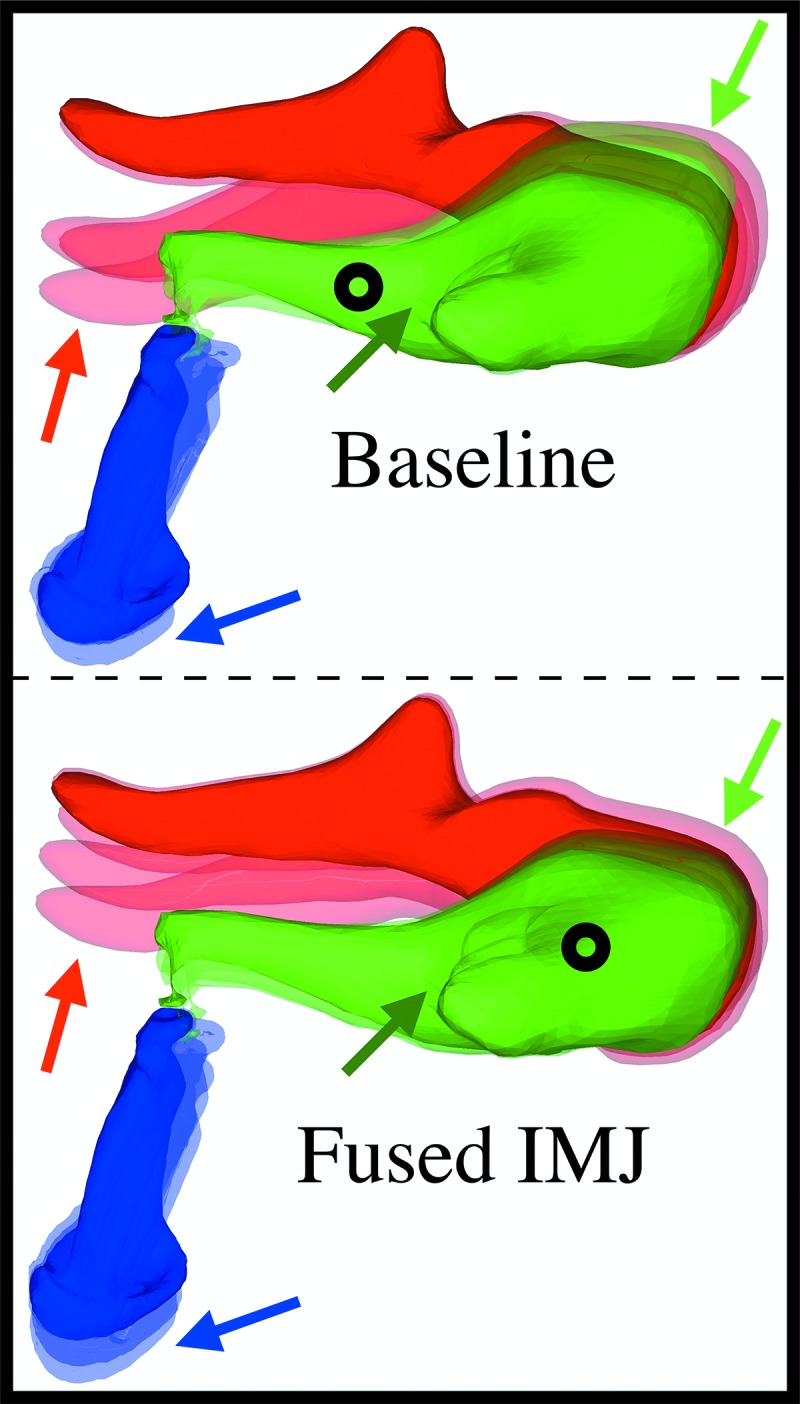
3D motion of the ossicles in a representative specimen. The reconstructed 3D movement of the ossicles (transparent overlays) is shown on top of the resting position (opaque images) in the baseline (top) and IMJ fused (bottom) cases. Note the changes in umbo motion (red arrows), incus-body motion (green arrows), and stapes-footplate motion (blue arrows). The black circles indicate the visually approximate location of the center of rotation of the incus in each case. Full animations of the motion are available online and should be viewed for a full understanding of the 3D motion (Mm. 1 and Mm. 2).

This calculation is performed at each measured time step to obtain the set of rigid-body motion components, and the overall time-dependent motion is reconstructed for each ossicle using a simple trapezoidal integration scheme. No assumptions about the mobility of the ossicular joints are made, since the motion of each ossicle is calculated independently without any kinematic constraints. The calculated motions are then applied to a solid model of human ossicles reconstructed from a *μ*CT scan (see Fig. 8).

### G. Computational models

**FIG. 9.**
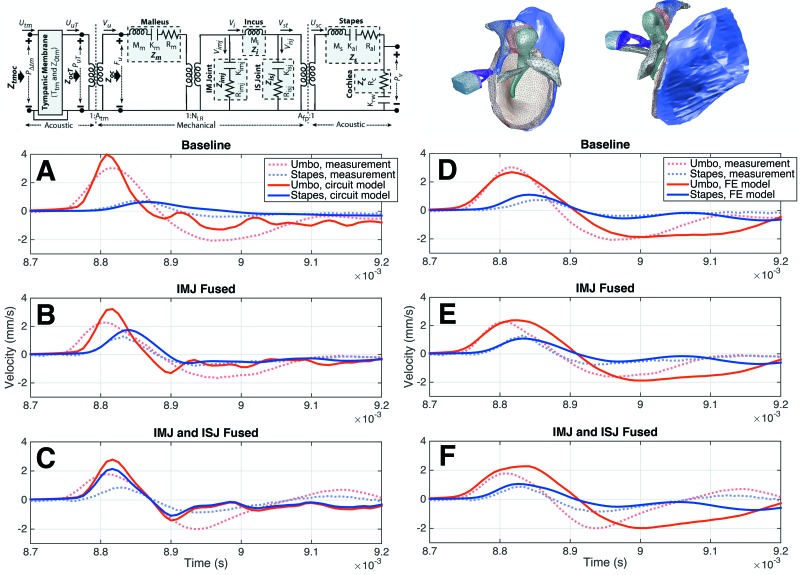
Computational models and their predictions. The circuit model (left) and FE model (right) are shown at the top. For more details about the models, see O'Connor and Puria (2008) and O'Connor *et al.* (2017). The umbo (red) and stapes (blue) velocity predictions (solid lines) of the circuit (A)–(C) and FE (D)–(F) models are shown along with the experimental velocities (dotted lines) from a representative specimen in the baseline [(A) and (D)], IMJ fused [(B) and (E)], and IMJ and ISJ fused [(C) and (F)] cases.

The results of the experimental measurements are compared to predictions by two previously developed computational models (see Fig. 9): an analytical circuit model (O'Connor and Puria, 2008) and an FE model (O'Connor *et al.*, 2017).

#### 1. Circuit model

The circuit model treats the TM as a transmission line, and the ossicles and cochlea as a series of lumped elements. Although O'Connor and Puria give a range of parameter values based on fits to two different sets of four and 12 ears, for the purposes of this study the most general set of parameters fit to the mean of all 16 ears was chosen. All model calculations are performed using Matlab. The model is solved for the umbo and stapes velocity in the frequency domain using an input pressure of 1 Pa at the TM. The frequencies are chosen to correspond to the frequencies in a fast Fourier transform (FFT) of the measured pressure impulse from the experiment. The resulting velocities are then convolved with the measured pressure signal by multiplying the two, and converted into the time domain using an inverse FFT to obtain the model response to the exact acoustic impulse used in the lab. The fused-joint cases are modeled by removing the elements corresponding to the joints, which each consists of a path to ground through a series capacitor and resistor.

#### 2. FE model

The FE model is based on geometry reconstructed from a *μ*CT scan, and consists of the external ear canal, TM, tympanic annulus, ossicles, joints, and supporting ligaments. The joints are modeled as homogeneous viscoelastic materials. The middle-ear cavity is not modeled. Material properties and boundary conditions are chosen to match the values given by O'Connor *et al.* (2017), and the FE model is solved using the COMSOL (version 5.2a) software package. The model is solved in the frequency domain at 1000 frequencies, evenly spaced from 1 Hz to 60 kHz, and the umbo and stapes velocities in the stapes-normal direction are extracted. The time-domain response to the measured acoustic impulse is then calculated in Matlab using the same method described for the circuit model. Simple linear interpolation is used to obtain the velocities at the FFT frequencies. To simulate the fused joints, the Young's modulus of the joints is set to match bone (*E* = 14 GPa).

## III. RESULTS

### A. Impulse transmission in the baseline case

Figure 4 shows a summary of impulse transmission through the ossicles in the baseline (unaltered) case for a representative specimen (TB13). The dashed black line shows the pressure at the location of the TM, while the solid lines show the velocity profiles (in the piston direction) of various points throughout the ossicular chain. The pressure measured in the middle-ear cavity is around an order of magnitude smaller than the pressure at the TM and is therefore not shown. A TM delay of 37.7 *μ*s is immediately evident from the spacing between the peak of the acoustic pressure and the peak of the umbo velocity curve (in red). While this varies by specimen, the mean TM delay across all specimens is 50.7 *μ*s with a standard error of 3.7 *μ*s. The velocity at the malleus head (magenta) shows a small delay relative to the umbo, but is also reflected about the *x* axis (i.e., it moves in the opposite direction) and has a smaller peak amplitude, indicating a seesaw-like motion of the malleus. A noticeable delay is evident between the malleus head and incus body (green), corresponding to delay in the IMJ. The incus-lenticular-process velocity (cyan) is reflected about the *x* axis again, indicating a seesaw-like motion of the incus as well. The velocity at the stapes crus (blue) closely matches the incus lenticular process, showing no obvious delay or change due to the ISJ in this specimen, though in some cases the ISJ effect is more notable. In this sample, the piston-direction velocity of the incus body slightly trails the velocity of the stapes due to a small component of gliding motion shown by the 3D motion reconstruction summarized in Sec. III E. The average ossicular delay (time between the peak umbo and stapes velocities) across all samples is 49.1 *μ*s, with a standard error of 4.9 *μ*s. Finally, the velocity measurement at the cochlear promontory (gray) shows that the velocities measured on the ossicles are far above the experimental artifact and noise floor of the measurement system.

### B. Effects of joint fusion in a representative sample

Figures 5(A)–5(C) show the velocity profiles at the five selected points on the ossicles, in various states of fusion (baseline, IMJ fused, and both joints fused), for a representative sample (TB13). Three main effects are evident. First, as the joints are progressively fused, the peak umbo velocity (red lines) is reduced, while the TM delay, as measured by the location of the peak umbo velocity, is largely unaffected. Second, fusing the IMJ eliminates the delay between the velocities measured on either side of the joint at the malleus head (magenta) and incus body (green). Due to the locations of the measurement points relative to the complex 3D motion of the ossicles (described later), and the locations of the measurement points relative to the axis of rotation of the malleus–incus complex, the curves do not follow each other exactly, with the point on the incus body moving more than the point on the malleus head. Importantly, though, the velocities peak at the same time as expected for a fused IMJ. Third, the stapes-crus velocity (blue) both increases in peak amplitude and is shifted earlier in time, following the elimination of the delay across the IMJ. The width of the main stapes-velocity profile is also reduced.

Since the goal of this work is to understand sound transmission through the ossicles (e.g., from the umbo to the stapes), the raw velocities are normalized to the umbo-velocity profile as described earlier. These results are shown in Figs. 5(D)–5(F). The umbo- and stapes-velocity profiles (red and blue) are additionally labeled with bars showing the calculation of the peak amplitude (dashed line) and FWHM (solid line). For the representative case shown, the umbo-velocity width is largely unaffected by joint fusion (the amplitude is exactly 1 due to the normalization). The normalized stapes-velocity peak amplitude increases by a factor of 2.0, and the width decreases by a factor of 0.87 from the baseline case to both joints fused.

**FIG. 10.**
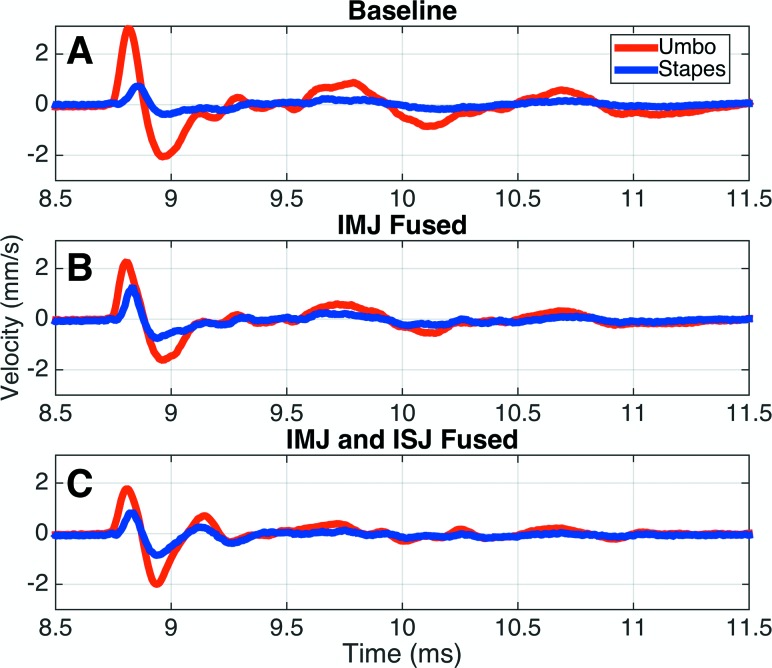
Effect of joint fusion on ringing. The umbo and stapes velocity curves for the representative specimen in Figs. 4 and 5 are shown over a longer time window in order to observe the effects of ringing. Note that as the joints are fused, the amplitude of the umbo ringing response diminishes while the stapes response increases. The response is shown for the baseline case (A), and the cases with the IMJ fused (B) and both joints fused (C).

Finally, as the sample proceeds from the baseline to a fully fused condition, the stapes displays a progressive increase in the level of ringing after the onset of the initial acoustic impulse, while the umbo does the opposite (see Fig. 10). In the baseline case, the initial umbo-velocity peak is followed by a large negative peak that roughly returns the umbo to its original position. After this, a ringing behavior with a period of about 1 ms is seen. The stapes, on the other hand, has a small negative recovery, but the prolonged ringing behavior is largely absent. Fusing the IMJ slightly reduces the amplitude of the ringing in the umbo response without noticeably changing the stapes response. In the fully fused case, however, both the umbo and stapes show the initial peak and recovery followed by a single additional ringing cycle with a much shorter period.

### C. Response to positive and negative impulses

Figure 6 shows the normalized velocity response at selected points throughout the ossicles to both the positive and negative pressure impulses generated during the measurements in a representative specimen (TB01). The response to the negative pressure [Figs. 6(D)–6(F)] is plotted on an inverted y-axis to facilitate comparison. Qualitatively, no difference is seen between the two sets of curves in any of the fusion cases, suggesting that the middle ear has a symmetric response to positive and negative pressures, at least in the range of pressures measured in this experiment (∼+20 to −20 Pa).

### D. Average effects of joint fusion across all samples

Table I summarizes the average changes in the normalized stapes-velocity profile after fusion of either or both of the IMJ and ISJ. The *p*-values are calculated using a 1-sided Student's *t*-test, and are highlighted in bold when significant at the *p* < 0.05 level. For one specimen, the experiment was halted after measuring the fused-ISJ-only case, resulting in the discrepancy in the numbers of specimens (the IMJ is fused first in five specimens and the ISJ is fused first in four specimens, but only eight specimens have both fused). Figure 7 highlights the most important results. The prototype impulse (dotted black line) has amplitude of 1 and an arbitrary width. The curves resulting from applying the mean change from fusing one or both joints are plotted along with the standard error for each case. Fusing just the IMJ has the largest effect (solid red curve), with a mean amplitude change of 2.4 and width change of 0.77, both significant (*p* = 0.033 and *p* = 0.021, respectively). Fusing both the IMJ and ISJ has a slightly smaller effect (solid green curve), with a mean amplitude change of 1.8 and width change of 0.78, again both significant (*p* = 0.0057 and *p* = 0.011, respectively). The higher levels of significance in this case are reflective of the larger sample size for the both-joints-fused case, since nearly all bones (*N* = 8) are tested in this state but only around half have just the IMJ or the ISJ fused. Fusing just the ISJ results on average in an insignificant change in both peak amplitude and width (solid blue curve).

Table II (second column) shows the average effects of joint fusion on the total ossicular delay, defined as the time (in *μ*s) between the peak umbo velocity and peak stapes velocity. Fusing the IMJ, ISJ, and both joints reduces the average delay from 49.1 to 36.6 *μ*s, 39.6 *μ*s, and 26.0 *μ*s, respectively.

**TABLE II.** Summary of measured and calculated ossicular delays (in *μ*s). The ossicular delay, defined by the time from peak umbo velocity to peak stapes velocity, is shown for each fusion case. Experimental results (both the average and standard error) and model results are shown.

State	Experiment	Circuit Model	FE Model
Baseline	49.1 ± 4.9	58.3	25.0
IMJ Fused	36.6 ± 4.3	25.0	8.3
ISJ Fused	39.6 ± 10.4	41.7	16.7
IMJ + ISJ Fused	26.0 ± 4.3	0.0	0.0

### E. 3D motion reconstruction

Figure 8 shows the reconstructed 3D displacement of a representative specimen (TB13) in the baseline (top) and fused-IMJ (bottom) cases, amplified by a factor of 7500 for visualization. The resting positions are shown by the opaque ossicles, while the transparent images represent the positions of the ossicles at evenly spaced snapshots in time. In both cases, the motion of all ossicles is predominantly in the stapes-normal direction, though other components contribute as well. There are three main differences between the motions of the baseline and fused-IMJ cases. First, the range of umbo displacement is much greater in the baseline case (red arrows). Second, the incus body drifts relative to the malleus head in the baseline case, but this motion is eliminated in the fused-IMJ case (light-green arrows). The entire malleus–incus complex also rotates more about an axis parallel to the long axis of the malleus in the fused case. This is most clearly seen by the change in motion of the short process of the incus in the fused case (dark-green arrows). In comparison to the baseline case (Fig. 8, top), the apparent incus center of rotation (black circles, approximated visually from animations) shifts superiorly for the fused-IMJ case. Finally, the stapes-footplate motion is larger in the fused-IMJ case, as seen by the greater range of displacement in the stapes-normal direction (blue arrows). The full animations of these cases as well as the both-joints-fused case are available online (Mm. 1, Mm. 2, and Mm. 3).

**Mm. 1.** Animation of reconstructed motion of the ossicles in the baseline case. The malleus, incus, and stapes are show in red, green, and blue, respectively. The stimulus pressure is shown above, along with a red circle indicating the time at each frame. This is a file of type “mp4” (189 KB).

**Mm. 2.** Animation of reconstructed motion of the ossicles as in Mm. 1, but for the fused-IMJ case. This is a file of type “mp4” (207 KB).

**Mm. 3.** Animation of reconstructed motion of the ossicles as in Mm. 1, but for the fused-IMJ and -ISJ case. This is a file of type “mp4” (96 KB).

### F. Computational-model predictions

The predictions of both the circuit and FE models for the change in stapes velocity relative to umbo velocity resulting from joint fusion are summarized in Tables II and III (note that the delay summarized by Table II is calculated according to the relative locations of the peak velocities rather than the onset of the curves). In general, the circuit model overestimates the amplitude and width changes, while the FE model fails to predict a meaningful change in either for all fusion cases. Overall, however, the models qualitatively match the experimental results much better than would be expected from the quantitative metrics alone, though discrepancies in certain aspects are notable.

**TABLE III.** Summary of model predictions for changes in stapes velocity after joint fusion (dimensionless).

	Stapes velocity **amplitude** ratio		Stapes velocity **width** ratio	
Joint(s) fused	Circuit Model	FE Model	Circuit Model	FE Model
Baseline → IMJ	3.316	1.110	0.7119	1.010
Baseline → ISJ	0.8768	1.059	1.293	0.9830
Baseline → IMJ + ISJ	4.7132	1.128	0.5946	1.054
IMJ → IMJ + ISJ	1.4215	1.016	0.8353	1.020
ISJ → IMJ + ISJ	5.375	1.065	0.4598	1.072

Figure 9 shows a comparison between the circuit [Figs. 9(A)–9(C)] and FE [Figs. 9(D)–9(F)] model predictions and the measured responses of a single representative specimen (TB13). Qualitatively, both models show a surprisingly good agreement with the experimental results, especially in the baseline cases. The circuit model [Fig. 9(A)] reproduces the stapes-velocity profile in the baseline case with good accuracy, qualitatively matching the amplitude and delay, though the model predicts a slightly wider curve than was measured. The umbo-velocity prediction is also qualitatively similar to the measurement, though it has a higher peak amplitude and smaller width. The FE model [Fig. 9(D)] accurately reproduces the shape and amplitude of the umbo-velocity curve. The stapes curve is also qualitatively similar to the experimental result, though the model curve is shifted earlier in time.

The circuit model is also surprisingly accurate in its prediction of the change in stapes velocity with the IMJ fused [Fig. 9(B)]. It qualitatively reproduces the experimentally measured increase in stapes-velocity peak amplitude as well as the change in delay. The umbo-velocity model prediction also follows the reduction in peak amplitude seen in the measurement. The circuit-model prediction fails in the both-joints-fused case [Fig. 9(C)], however. It does not reproduce the residual delay of 26 *μ*s between the umbo and stapes from the experiment, nor does it correctly predict the change in the ringing behavior of the ossicles.

The FE model is also successful at predicting various aspects of the experimental results in the fused cases, though differently from the circuit model. After fusing the IMJ, the FE model overestimates the width of the umbo velocity but correctly reduces the peak amplitude [Fig. 9(E)]. The model's stapes velocity matches the delay and amplitude in the fused-IMJ case, though this is more a result of the change in the experimental curve, since the model's stapes velocity is essentially unaffected by fusing the joint. After additionally fusing the ISJ [Fig. 9(F)], the FE model again predicts no change in the stapes velocity, overestimates the width of the umbo-velocity curve by a large margin, and fails to reproduce the residual delay between the umbo and stapes found in the experimental results.

## IV. DISCUSSION

### A protective middle ear?

A.

#### 1. The middle-ear filtering effect

The results of this experiment show that fusing the ossicular joints causes a significant increase in the stapes velocity resulting from an impulsive acoustic stimulus. Thus, it appears that the mobile joints do indeed provide a filtering effect that reduces peak stapes velocity from these stimuli. The experiment additionally shows that the mobile joints introduce delay and cause a broadening of the transient stimulus as it progresses from the umbo to the stapes. The joints may achieve this filtering effect at least in part through frequency dispersion due to the nonlinear properties of the synovial joint capsules (Hron *et al.*, 2010). As seen in Fig. 10, the joints also provide a filter to dampen the negative peak and remove the ringing artifacts seen at the umbo before they reach the stapes. This is likely due to a straightforward damping effect arising from the viscosity of the fluid inside the joint capsules. Interestingly, though the ISJ is a smaller joint and less commonly thought of as a major source of damping, it apparently plays an important role in filtering the ringing as seen from the change in the stapes velocity curves (blue) between Figs. 10(B) and 10(C).

#### 2. Noise-hazard implications from impulsive stimuli

One might argue that since the cochlea itself is already a highly dispersive medium, the additional dispersive effects of the middle-ear joints should not matter. The goal of this study, however, has been to investigate the transformation of the signal that arrives at the input to the cochlea, and the two main measured effects of the joints on impulse transmission—peak-amplitude reduction and pulse broadening—are tied to the potential for the impulse to damage sensitive cochlear structures. Though an accurate algorithm for assessing the hazard associated with acoustic impulses has been elusive, it is generally agreed upon that the hazard depends on a combination of the number of exposures, peak amplitude, and sharpness of the stimulus (Price, 2007). Furthermore, Patterson (1991) concluded that an overall 3 dB reduction in sound-exposure level offsets a doubling of the number of impulses. If this so-called “equal energy hypothesis” is true, then it follows that the flexible ossicular joints increase the number of tolerable impulses since they reduce the peak stapes velocity by a factor of about 6 dB.

#### 3. Clinical implications

The findings that the ossicular joints may provide a protective filtering effect have important implications for clinical scenarios in which joint flexibility is altered. In the case of an interrupted ossicular chain, a prosthesis is commonly used to bridge the gap and improve hearing. Most prostheses are essentially rigid struts that connect the TM, malleus, or incus directly to the stapes capitulum or footplate and bypass one or both of the ossicular joints. Despite this, a well-positioned prosthesis can restore hearing to essentially normal levels (Gottlieb *et al.*, 2016). In removing the protective effect of the joints, however, the prostheses may increase the patient's susceptibility to harm from acoustic impulses, resulting in slow but progressive hearing loss from accumulated damage. This could lead to increased interest in new prosthesis designs that incorporate joint-like flexible elements. Further study is necessary in order to determine the validity of this hypothesis.

A similar effect could help explain the relatively high prevalence of hearing loss in patients with rheumatoid arthritis (RA), a disease that typically targets synovial joints. Colletti *et al.* (1997) have found that 40% of RA patients in their study had abnormal tympanograms, indicating a stiffened ossicular chain. They hypothesize that, although the change in middle-ear mechanics due to RA did not significantly impair sound transmission, it might reduce protective mechanisms toward static pressure. Others have argued that the hearing loss comes from a dual effect of subclinical middle-ear changes coupled with biochemical changes in the inner ear (e.g., Özcan *et al.*, 2002; Ikiz *et al.*, 2007). The results of the present study support the idea that a stiffening of the ossicular joints because of RA could lead to progressive sensorineural hearing loss due to reduced protection not only from static pressures but also from damaging acoustic impulses. Since degradation and stiffening of the ossicular joints has been found to increase as a function of aging in the general population as well (Belal and Stewart, 1974; Etholm and Belal, 1974), the contribution of this effect to overall age-related hearing loss may be important and warrants further study.

### B. Factors in middle-ear impulse transmission

The idea that the middle ear transforms and adds delay to signals as they propagate to the cochlea is not new (e.g., Puria and Allen, 1998; Parent and Allen, 2007; O'Connor and Puria, 2008), but the source and function of these transformations and delays has been a topic of debate. This experiment allows these effects to be broken down into contributions from four main components: the TM, the IMJ, the ISJ, and the flexibility of the ossicles themselves (primarily the manubrium of the malleus).

#### 1. The TM

Extracting the effect of the TM alone from this experiment is complicated by the fact that using the umbo velocity relative to the acoustic input as a proxy invariably includes the downstream effects of the ossicular chain. Therefore, it is not appropriate to immediately credit the TM for changes in the umbo velocity due to joint fusion. Still, it is possible to gain an understanding of the TM in the baseline (fully mobile) case, and hypothesize as to the cause of changes resulting from fused joints.

In the baseline case, the delay from peak input pressure to peak umbo velocity ranges from 37.7 to 62.7 *μ*s, with an average of 50.7 *μ*s. This is slightly higher, though still in the range of the estimates of O'Connor and Puria (2008) using their circuit model, which puts the TM delay at 32.1 *μ*s with a range of 17.6 to 75.5 *μ*s. In general, the human TM delay seems comparable to what has been estimated for cat (40 *μ*s, from Puria and Allen, 1998) and higher than what has been measured in a gerbil [5–15 *μ*s from Milazzo *et al.* (2017); or 9 *μ*s from de La Rochefoucauld *et al.* (2010)], though it is likely that the shorter delay in a gerbil has much to do with the smaller size of the gerbil TM and middle-ear structures.

In this experiment, the response at the umbo is typically not only delayed but also broadened, an effect that is noted in all three states of fusion as well. This broadening contributes to the overall change in the impulse shape by the time it reaches the stapes. This result contrasts with the findings in a gerbil of Milazzo *et al.* (2017), who have noted that the shape of the acoustic input is reproduced with very high fidelity at the umbo, even when presenting a click with a duration on the order of 0.05 ms, which is about half the width of the impulses used in this experiment. As with the differences in TM delays between humans and gerbils, this discrepancy is likely due to the relative sizes of the middle-ear structures between the two mammals. Human middle ears, with their larger and more massive ossicles and TM (the human malleus–incus complex is more massive by more than a factor of 10), have a lower resonance frequency and thus respond more slowly to transient stimuli. Presumably, given a wide-enough impulse, the human umbo response would also reproduce the acoustic input with high fidelity. Conversely, given a short-enough impulse, even the gerbil would show some pulse-spreading at the umbo.

Fusing the joints in this experiment has little effect on the delay or width of the umbo-velocity profile, but does typically result in a reduction of peak umbo velocity. This can be attributed to the increase in the middle-ear input impedance that results from stiffening the ossicular chain. This effect is well known and used clinically in the form of tympanometry, which can diagnose stiffened ossicular chains arising from a variety of diseases (Margolis *et al.*, 1999). Since, on average, joint fusion results in a net increase in stapes velocity, this change is clearly offset by the increases in transmission efficiency from the umbo onwards.

#### 2. The IMJ

In this experiment, fusing the IMJ has a significant effect on both the peak amplitude and width of the stapes-velocity curve. It also accounts for the majority of the change in stapes velocity in the fully fused case, as seen in the final row of Table I. This, along with the obvious delay between the malleus head and incus body seen in Figs. 4 and 5, suggests a mobile IMJ. This is consistent with numerous previous results that have shown a mobile IMJ that causes transmission loss in the case of steady-state acoustic stimulation (e.g., Offergeld *et al.*, 2006; Gerig *et al.*, 2015; Willi *et al.*, 2002) or allows for umbo motion even when the rest of the ossicular chain is immobilized (Nakajima *et al.*, 2005).

The current study sheds new light on this in two ways. First, it yields additional evidence that the mobility of the IMJ is relevant to realistic acoustic inputs such as gunshot-like impulses. Second, the 3D measurements and velocity reconstructions provide some insight into the possible mechanism of the increase in stapes velocity resulting from a loss of IMJ mobility; see Fig. 8, for example (and Mm. 1 and Mm. 2, online). In the normal, fully mobile state, the rocking motion of the malleus is transferred to the incus through the saddle shape of the IMJ. This results in a rocking motion of the incus, but the shape and flexibility of the joint result in much of the incus motion being localized near the incus body, and it is possible to imagine an axis of rotation passing through the middle of the incus long process (note the black circles on Fig. 8). Fusing the IMJ, however, removes the effect of the saddle shape, which forces the incus motion to follow the malleus and moves this imaginary rotation axis toward the IMJ. This results in a longer effective lever arm of the incus and more motion at the stapes. Of course, fusing the IMJ also induces a greater degree of “twisting” motion (motion other than simple in-plane rocking), but the mobility of the ISJ transforms this into piston-like motion of the stapes, as discussed next in Sec. IV B 3.

#### 3. The ISJ

Although the role of the ISJ appears to be less important for sound transmission than that of the IMJ, the results of this experiment show an interesting split in ISJ behavior. Apparently, the ISJ plays a different role depending on whether the IMJ is mobile or fused. When the IMJ is mobile, fusing the ISJ has an insignificant effect on stapes velocity, as reflected in the “ISJ Fused” case in Fig. 7 and Table I. This is similar to the conclusions of Elpern and Elbrond (1966), who find that bridging an interrupted ISJ with rigid acrylic restores sound transmission to the stapes to normal (pre-interruption) levels, measured in the frequency domain up to 4 kHz. In a more recent study, Alian *et al.* (2013) find that stiffening the ISJ with cyanoacrylate adhesive causes a small decrease in displacement at both the umbo and the stapes, though the decrease in displacement is only statistically significant between 400 and 1000 Hz, with a mean value of 6 dB. Importantly, they find that it produces little change in their relative motion since both the umbo and stapes displacement magnitudes are affected by the same amount (they do not report any phase measurements).

**FIG. 11.**
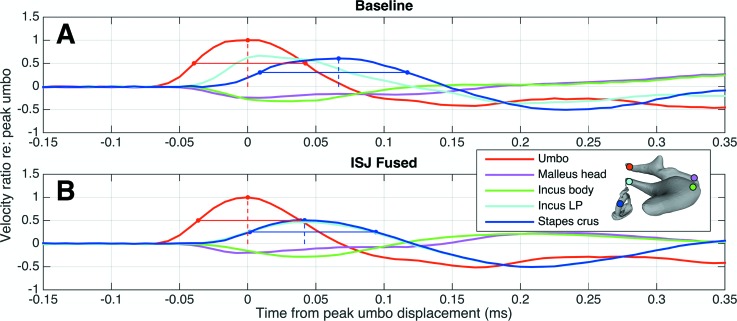
An outlier case of ISJ mobility. The velocities (normalized to the peak umbo velocity as in Fig. 5) at five points on the ossicles in a different specimen are shown in the baseline (A) and ISJ fused (B) cases. Although most specimens show little mobility in the ISJ, this and one other specimen demonstrate a clear delay across the ISJ, as seen by the motion at the lenticular process (Incus LP, cyan) and stapes crus (blue) in the baseline case. This relative motion is no longer evident after the ISJ is fused.

Evidently the mobility of the ISJ normally plays little role in sound transmission. Indeed, in all but two of the nine temporal bones tested in this experiment, no noticeable delay or amplitude change is observed between the points measured on the incus lenticular process and the stapes crus. Interestingly, in the two cases in which the ISJ has an obvious effect, fusing it appears to affect the velocity of the incus lenticular process more than the velocity of the stapes, suggesting that while a loose ISJ may allow the incus lenticular process to flap around, it still transfers a similar magnitude of vibration to the stapes (see Fig. 11).

When the IMJ is already fused, fusing the ISJ counterintuitively results in a decrease in stapes velocity relative to the IMJ-only case, by a factor of 0.73 on average [see Table I; also note, for one temporal bone, how the fused-IMJ curve in Fig. 6 (blue) is slightly larger than the case with both joints fused]. On first glance this is puzzling, particularly since as just noted fusing the ISJ in isolation appears to have a minimal effect. To explain this phenomenon, it is helpful to think in terms of the actual 3D motion of the ossicles and the alignment of the incus and the stapes. In the fully mobile case, the rocking motion of the malleus is transferred to a rocking motion in the incus. The incus long process and pedicle, however, do not form a line perpendicular to the stapes footplate. Rather, there is a small angle of approximately 23° (Stuhlman, 1937) such that when the incus rocks, the stapes moves primarily in the footplate-normal direction at the location of the footplate, but there is a conspicuous side-to-side rocking motion of the stapes head as well (see Fig. 8 and Mm. 1).

Fusing the IMJ constrains the relative movement between the malleus and incus and therefore changes the motion of the incus (as noted in Sec. IV B 2). Since the relationship between the incus long process and the stapes is unchanged, however, this new mode of incus vibration is similarly transferred into a rocking at the stapes head which results in a displacement largely in the footplate-normal direction at the footplate. Fusing the ISJ forces the stapes motion to exactly follow the incus lenticular process, which causes the stapes to move even more predominantly in the footplate-normal direction (see Mm. 3). It also, however, constrains the kinematics of the entire ossicular chain and reduces the total motion that is transferred to the stapes in the first place. This effect is presumably present when fusing the IMJ after the ISJ as well, though it is not observed because the amplitude-increasing effect of fusing the IMJ is significantly larger.

#### 4. Bone flexibility

If the ossicular joints were the only sources of flexibility in the ossicular chain, fusing them should result in a complete elimination of the delay between the umbo and the stapes. This has not been found to be the case in this experiment, as a residual delay of 26.0 *μ*s is still seen even in the fully fused case [Figs. 5(C), 5(F), and Table II]. This is similar to the findings of Hato *et al.* (2003), who noted a residual delay of 35 *μ*s after fusing both joints. They attribute this to elasticity of the middle ear resulting from tension in the supporting ligaments and tendons. While this certainly contributes, the results from the current experiment suggest that flexion of the ossicles themselves, and particularly the manubrium of the malleus, is largely responsible for the remaining ossicular delay. This is most clearly seen by looking at the locations of the peak velocity at the umbo and the malleus head. In the representative example in Fig. 5, the delay between these two curves is negligible in the fully mobile case, but increases after the IMJ is fused, until in the fully fused case it accounts for the entirety of the total ossicular delay. The specimen shown in Fig. 6, which had particularly stiff joints throughout the experiment, shows a similar delay between the umbo and malleus head in all states of fusion. This result can be explained by treating the manubrium, which is a relatively long and thin structure, as a beam pinned at one end (at the joint) in the fully mobile case. A force at the other end (the umbo) thus causes a rigid rotation with little to no bending. In the case of a stiff or fused IMJ, the end is instead fixed, resulting in a cantilever beam that bends along its length. A similar bending of the manubrium has been noted in a gerbil (de La Rochefoucauld *et al.*, 2010). The prediction of the relative contributions of bone and joint flexibility to sound transmission are also particularly good tests for computational models of the middle ear, which are discussed next in Sec. IV C.

### C. Potential for computational-model improvements

The general benefits and drawbacks of using a circuit rather than an FE model in middle-ear studies are commonly known—a circuit model is computationally inexpensive and can give accurate results, though they can be limited to somewhat abstract quantities that must be interpreted as physiological responses. On the other hand, FE models can provide full 3D predictions of middle-ear behavior, at the cost of dramatically longer computation times. Despite these factors, though, it was unclear which model would best be able to predict the time-domain behavior measured in this experiment.

The two models tested in this study show surprisingly good qualitative agreement with the representative experimental results they are compared to, without any adjustments to the original parameter values. Still, the discrepancies between the predicted quantitative changes in stapes velocity and the experimental averages suggest room for improvement in both models. Interestingly, the two models trend in opposite directions—while the circuit model overestimates the role of joint fusion on stapes peak amplitude and width, the FE model is very insensitive to changes in the joint parameters. Further, while the circuit model was generally more successful at predicting the falling edge of the velocity impulse, the FE model was better at predicting the onset and rising edge.

The failure of the FE model to predict the effects of joint fusion points to the need for a closer look at the joint properties. At first glance, it seems that the joints are modeled as being too stiff, since stiffening them further has little effect. In addition to this, though, the model might benefit from a more detailed and accurate description of the joints. Currently, the joints are modeled as a soft, homogeneous material that fills the spaces between the ossicles. This is a commonly used modeling strategy (e.g., Koike *et al.*, 2002; Sun *et al.*, 2002; Gan *et al.*, 2004; Zhao *et al.*, 2009) that is relatively cheap computationally and straightforward to define in a finite-element framework. Physiologically, though, both joints instead consist of synovial-fluid-filled capsules surrounded by ligaments. This system displays nonlinear behavior due to the non-Newtonian nature of synovial fluid, which is not captured by using a homogeneous model, even if viscoelastic properties are included. Some effort has been put into building models of the joints that include the fluid effects (e.g., Zhang and Gan, 2011), but more work remains in incorporating these into full models of the middle ear.

Despite their differences, both models are unable to reproduce the residual delay present between the umbo and stapes, even when both joints are fused. In the circuit model, the malleus and incus levers are represented as an ideal transformer. This would need to be modified to include more complex non-ideal elements incorporating stiffness and mass to produce delay even after removing the joints. With a Young's modulus (14 GPa) similar to cortical bone and measured malleus (2390 kg/m^3^) and incus (2150 kg/m^3^) densities (Sim and Puria, 2008), flexibility of the malleus and incus bones is implicitly incorporated into the FE model. The lack of sufficient delay there suggests that the effective Young's modulus of the bones in the FE model may need to be lowered.

### D. Benefits of this measurement technique

In this study, the effects of ossicular-joint flexibility in the human ossicular chain have been investigated using direct measurements of the transient response of the middle ear in the time domain. This allows for the direct observation of the various sources of delay in this system, as well as the evolution of a realistic impulse as it propagates through the middle ear.

Further, the use of a 3D measurement system has enabled the computation of the full, complex motion of the ossicles, without the need for the various simplifying assumptions required when these measurements are performed using one-dimensional (1D) methods. Although the analysis in this study is later restricted to the stapes-normal direction, this has only been done after confirming that this is indeed the dominant component of the motion and that analysis done on this motion component would be sufficient to capture the effects under investigation.

Using the 3D vibrometer also makes it possible to measure the vibration response of points throughout the ossicular chain, from the umbo to the stapes crus, without rotating the specimen or requiring the laser to be moved manually, which reduces the potential for introducing error and uncertainty when consolidating measurements into a single reference frame. While it is possible to focus a 1D LDV on all of the measured points without changing frames, the laser (and therefore, measurement) axis would need to be almost perpendicular to the primary motion component. Without an *a priori* assumption of the ossicular vibration modes, then, it would be difficult to estimate the true magnitude of vibration at the measurement locations.

### E. Limitations and future directions

The goal of this study has been to investigate the role of ossicular-joint flexibility on the transmission of potentially harmful acoustic impulses through the middle ear. Although these measurements are successful, it is important to note that the amplitude of the stimulus impulses (peak pressure around 20 Pa) is well below what would arise from a standard weapon discharge (pressures on the order of kPa). Therefore, any effects arising from the nonlinearity of the middle ear at high sound pressures have not been captured, but would presumably be additive. In addition, the impulses generated in this experiment have a relatively short duration, roughly equivalent to what would be seen from a rifle (Price, 1986). Blasts from other weapons, such as cannons and improvised explosive devices, have longer durations. Nevertheless, these results provide insights into the behavior of these joints that can be extrapolated to the other cases that have not been directly studied.

Another limitation of this study is the use of stapes velocity as a proxy for cochlear pressure. Although it has been demonstrated that this is a reasonable assumption (Puria *et al.*, 1997; Aibara *et al.*, 2001), it is impossible to determine the exact effect of joint flexibility on cochlear mechanics with these measurements. In the same vein, these measurements do not directly address whether the transformation of the impulses due to the joints changes their ability to damage inner-ear hair cells after the frequency-to-place mapping and dispersion by the traveling wave. Future experiments might investigate these questions by measuring cochlear pressure in human temporal bones, or by looking at hair-cell damage in response to transformed impulses in animal models. Computational-modeling approaches could also be used to study the behavior of the basilar membrane and the hair bundles in response to impulsive stimuli, leading to a more complete picture of the transmission and perception of these sounds.

## V. CONCLUSION

While the physiological existence of delay in the human middle ear has been well established, its physiological role has been debated. The present results suggest that middle-ear flexibility reduces the peak amplitudes of impulsive sounds and thus may serve to protect the sensory structures of the organ of Corti.
